# Regulation in ecological systems: an overview

**DOI:** 10.1007/s40656-025-00707-0

**Published:** 2025-12-08

**Authors:** Clarissa Machado Pinto Leite, Ítalo Nascimento de Carvalho, Jeferson Gabriel da Encarnação Coutinho, Charbel N. El-Hani

**Affiliations:** 1 Institute of Biology, Federal University of Bahia, Salvador, Brazil; 2 National Institute of Science and Technology in Interdisciplinary and Transdisciplinary Studies in Ecology and Evolution, Salvador, Brazil; 3 Bahia Federal Institute of Education, Science and Technology, Salvador, Brazil

**Keywords:** Dynamic stability, Feedbacks, Homeostasis, Ecological regulation, Control

## Abstract

The concept of regulation is used in biology to explain properties such as stability, robustness, and long-term persistence of biological systems. Regulation claims often focus, however, on the capabilities of these systems, rather than the mechanisms underlying them. In an attempt to produce a full-fledged theoretical account of regulation, a stricter concept has been proposed by Bich and colleagues based on the theory of biological autonomy. According to these authors, regulation is exerted by specialized subsystems that can change the constitutive regime of a system (enabling it to deal with a wider range of perturbations) but whose dynamics are not specified by the latter. In this paper, we present a brief survey on how regulation has been treated in biology and, more specifically, in ecology. We show how the ecological literature attributes regulatory powers either to organismic phenomena or to the propagation of perturbations through the network of relations in ecological systems. We were not able to find any study proposing a specialized subsystem dedicated to regulation in an ecological system. This raises doubts about the feasibility of a regulatory subsystem such as conceived by Bich and colleagues. Nonetheless, we argue that ecological systems may encompass more than just dynamic stability and feedback mechanisms, such that it can be worthy applying their concept to investigate regulation in these systems.

## Introduction

The concept of regulation is used in biology to explain properties such as stability, robustness, and long-term persistence of living beings. Recently, it has become a subject of growing interest among philosophers of biology (e.g., Bich et al., 2016; Winning & Bechtel, 2018; Bich et al., 2020; Bolton & Šustar, 2022; Gonzáles de Prado & Saborido, 2023). Although regulation has been widely applied to understand living systems, efforts to build a comprehensive theoretical account have been much more limited. Additionally, biological organisms deploy a variety of mechanisms and strategies to ensure their survival. The tendency to focus on the regulatory effects rather than on the nature of the compensatory responses has contributed to inaccuracies about the meaning of regulation. This focus has also reinforced ambiguous relationships with other associated concepts, such as control, homeostasis, feedback, and adaptation (Bich et al., 2016).

Most theoretical explanations about regulation in biological systems focus on the notions of homeostasis, feedback mechanisms, and dynamic stability (e.g., Jones, 1973; Hofmeyer & Cornish-Bowden, 1991; Hofmeyer & Cornish-Bowden, 2000; Heylighen & Joslyn, 2001; Heylighen, 2002). However, Bich and colleagues (2016) have proposed a fundamental distinction between two qualitatively distinct ways to achieve biological robustness: (1) dynamic stability and feedbacks, as a collective network response to perturbations performed by the constitutive regime of biological systems, and (2) biological regulation, which consists in a specific form of second-order control exerted by a specialized subsystem over the constitutive system involved in the production and maintenance of the components that fundamentally compose the organism. According to these authors, unlike dynamic stability and feedbacks (which correspond to a more elementary distributed control capacity), biological regulation requires a distinct architecture of functional relationships. More specifically, it requires the action of a dedicated subsystem whose activity is dynamically decoupled from that of the constitutive regime. 

They advance this distinction based on the theory of biological autonomy, which treats biological systems as organizationally closed and thermodynamically open (Moreno & Mossio, 2015).^1^ In their systematization of this theory in book format, Moreno and Mossio state that biological organization should be understood as a closure of constraints, i.e., as a mutual dependence network involving constitutive parts of the living systems that act as constraints. Constraints are local and contingent causes that reduce the degrees of freedom of the dynamics or processes on which they act (Pattee, 1972). They remain conserved at the time scale relevant to describe their causal action with respect to that process or dynamics. This means that they are not affected by the process under their influence at that time scale in the properties relevant to their causal action (Montévil & Mossio, 2015; Moreno & Mossio, 2015; Mossio & Bich, 2017). Closure of constraints involves, according to this theory, all constraints that participate in the production of at least one other constraint and are produced under the influence of at least one other constraint, i.e., by all constraints that are both enabling and dependent. This mutual dependence network among constraints establishes a constitutive regime, which can also be considered a first-order closure. These constraints contribute to determining the behavior of the system precisely by reducing the degrees of freedom of its internal processes and dynamics, which, in turn, generates new possibilities for the system as a whole due to the coordination of its parts.

Although other types of systems, such as chemical or physical ones, are also influenced by constraints, only biological systems realize – according to the theory – a closure of constraints, which confers their distinctive generative dimension as well as their intrinsic teleology. The intrinsic teleology of biological systems specifically means that their own activity is, in a fundamental sense, first and foremost oriented toward an end, namely, maintaining the conditions that allow the persistence of the system itself or, more precisely, of the organization it instantiates. In summary, organisms “are what they do” (Moreno & Mossio, 2015, p.104).

However, according to the theory of biological autonomy, biological organization, to be such, also requires regulation. The robustness of biological organisms is related to their capacity to self-maintain in stable conditions, despite continuous internal and external perturbations that are potentially deleterious, i.e., that threaten their self-maintenance. Regulatory capacities govern the transition toward a new viable situation as threatening perturbations impact the system, be it by countering the perturbations themselves or by establishing a new regime of constitutive organization. In terms of the theory, regulation is explained, then, in terms of a specific set of constraints that contribute to the maintenance of the organization only when its closure is being disrupted. Nevertheless, regulatory constraints are not subject to first-order closure because they exert their causal actions on changes in the constitutive constraints of the organization. Therefore, the theory argues that regulatory constraints should be understood as being subject to second-order closure, which allows us to distinguish the *regulator* subsystem from the *regulated* one (Bich et al., 2016).

This new definition of regulation is not trivial, and a better understanding of how it differs from current uses of the term is in order. Additionally, since it was originally developed to account for the properties of organisms, it is not yet clear whether (or how) it would apply to other biological entities, such as ecological systems. This question is particularly relevant given the growing disruptions that lead to the collapse of ecosystems worldwide. Understanding how ecosystems respond to disturbances is increasingly critical, and if the organizational framework provides new insights or allows for more detailed representations and interpretations, there is no reason not to leverage its theoretical promise.

Furthermore, the theoretical literature shows that discussions about regulatory mechanisms in living systems have intensified in recent years, as discussed above. Understanding the different forms of control in biological systems—how they emerged and what functions they serve—are central questions that have been typically addressed at the subcellular, cellular, or organismic level. Exploring these issues at higher levels of organization, such as ecosystems, can also provide valuable insights that feed back into these original investigations. In 2014, Nunes-Neto and El-Hani extended the organizational approach to ecological systems, establishing an organizational definition of ecological function (Nunes-Neto et al., 2014). In their original article the authors made use of a bromeliad phytotelma to exemplify an organizationally closed ecological system, providing the first systematic foundation for what would develop into the organizational theory of ecological functions. Subsequent theoretical advancements have progressively refined this framework by clarifying the incorporation of abiotic components as functional elements (El-Hani & Nunes-Neto, 2020); developing new arguments for the individuation of causal systems, including ecological cases (El-Hani et al., 2025); and addressing critiques concerning the individuation of organizationally closed ecological systems, the ascription of organizational functions to abiotic items, and the evolution of organizational functions through the co-option of organismal activities (El-Hani et al., 2024). Given that regulation plays a central role in the self-maintenance of living systems, it is relevant to explore whether second-order regulatory regimes, as described by Bich and colleagues (2016), can be identified in ecological systems.

In the following sections, we will first describe some theoretical accounts of regulation found in the literature, more specifically, those related to the notions of homeostasis, feedback mechanisms, dynamic stability, and adaptivity, as well as the proposal made by Bich and colleagues (2016) on the distinction between more elementary control capacities and biological regulation. Next, we will show how regulation is treated in ecology and argue for the importance of theorizing about regulation in ecological systems according to this distinction between different types of control capacity. We will also suggest that there may be more to ecological systems than dynamic stability and feedback mechanisms, such that it can be worthy applying the concept of regulation, as proposed by Bich and colleagues, to investigate regulation in such systems. In doing so, we hope to stimulate new investigations into cases of regulation (*sensu* Bich and colleagues) in ecological systems and the construction of models to account for it.^2^

## An overview of the concept of regulation in biology

### Homeostasis and feedback loops

The concept of homeostasis was proposed by Walter Cannon in 1929, inspired by the work of Bernard (1865, 1878), who explained the contribution of the organized parts of a living system to the conservation of its internal *milieu* despite the continuous variations occurring in the external *milieu* (see discussions in Moreno & Mossio, 2015; Mossio & Bich, 2014, 2017; Mossio et al., 2016). According to Bernard, biological systems are capable of self-determination because they contain distinctive aspects that are not displayed by other systems, such as physical systems or artifacts. His approach addresses these biological distinctive aspects without appealing to vitalist principles. Explaining the differences between natural laws, common to all phenomena, and *milieux*, as establishing local boundary conditions that determine how those laws operate within them, Bernard elucidated how different *milieux* can harbor qualitatively distinct phenomena without contradicting general physical laws. Bernard identified, accordingly, two fundamentally distinctive properties of the internal *milieu* of biological systems: its self-determination, as all components contribute to the realization of the conditions in which all other components can exist, and its ‘constancy’ or stability, even though continuous variations take place in the external *milieu*. These two properties express the intrinsic teleological dimension of living systems in Bernard’s view, since organisms are not only capable of compensating for external variations by means of internal modifications but also because the conservation of the internal *milieu* serves the main intrinsic goal of maintaining the specific internal conditions for the organism to exist (Mossio & Bich, 2017).

According to Bernard, the internal *milieu* of living systems shows constancy as a consequence of internal control or regulation since all organisms perform processes and activities subject to regulatory mechanisms, adapting their physiological processes on a continuous basis and thus maintaining their vital activities. Based on this idea, Cannon (1929) proposed that homeostasis refers to the ability of biological organisms to compensate for external disturbances through a coordinated physiological action made possible by the plasticity of their internal environment. In other words, homeostasis is a mechanism of stabilization that formalizes Bernard’s notion of conservation of the internal *milieu* (Mossio & Bich, 2014, 2017).

The influence that the introduction of the feedback loops concept exerted on homeostasis modeling is evidenced by the proliferation of models and applications using both concepts in first-order cybernetics in the 1940s and 1950s. This proliferation of applications, especially in the context of servomechanisms, has made cybernetics the general framework for the investigation of teleological and adaptive behaviors in a wide range of domains, including biology (Mossio & Bich, 2014, 2017). A feedback loop can be understood as a causal relationship between a sensor and an effector, controlled by a regulator capable of acting on the effector from a disruptive deviation detected by the sensor to activate a compensatory action (in this case, negative feedback). In negative feedback, the system’s response counteracts the influence of a stimulus, keeping the system in a particular state, whereas positive feedback amplifies changes in the face of the stimulus and may destabilize the system’s state.

Although its generality is a key reason for cybernetic treatment to be highly valued, it can also become a weakness when applied to account for biological self-determination (Mossio & Bich, 2017). While the cybernetic approach has the merit of proposing an explicit model of causal circularity, this model is not at work exclusively in biological self-determination, the distinctive aspect of living systems in relation to other natural systems and artifacts, as defended by Bernard himself. Homeostasis does not designate, then, a systemic capacity that sufficiently accounts for the distinctive regulatory capacity of biological systems because its extension is larger than, more general than, the domain of such systems. As Mossio and Bich (2014, 2017) argue, when described as a result of the action of stabilization mechanisms, homeostasis does not correspond to the regime of self-determination in living systems but rather to a general ability to compensate for environmental perturbations that can also be found, for instance, in artifacts. The capacity for homeostasis presupposes the existence of a system whose organization must, in certain circumstances, be kept stable. In turn, this stability is, in the case of living systems, closely related to their self-determination capacity. In itself, homeostasis does not capture the most distinctive generative dimension of biological organization, i.e., the fact that the components involved in feedback loops are not only stabilized but also produced and maintained by the very organization to which they belong. This is why the cybernetic approach misses the crucial dimension of biological intrinsic teleology, as it blurs its specificity in relation to the extrinsic teleology at work in artifacts.

Relevant sources dealing with regulation in the biological sciences, such as, for instance, Richard Jones’ ‘*Principles of Biological Regulation*’ (1973), mostly focused on the stability of living systems by appealing to homeostatic mechanisms and feedbacks. For Jones, regulation, when used to describe a property of a system, refers to the minimization of changes caused by disturbances as well as to the mechanisms by which this minimization is achieved. According to him, in a biological context, “homeostasis” and “homeostat” are terms used to convey this meaning of regulation that suggest the possibility of specifying those portions of the system that endow it with this property. Therefore, biological regulation is related to control mechanisms (i.e., feedbacks) that Jones attributed to homeostatic systems in the body, with the function of acting to minimize the effects of disturbing environmental factors. He also stated that this is a means by which organisms have achieved relative freedom from the constraints of their environments and, because of this, have acquired a greater variety of functions, not only those necessary to maintain homeostasis but also those allowed by increased environmental exposure. According to him, organisms have acquired not only one but a very large number of homeostats, each related to some physical aspect of their internal affairs or their behavior in relation to the environment. In addition, each of these homeostats has its own limits, within which it can maintain its integrity, and excursions beyond these limits are almost invariably fatal. Although highlighting the existence of aspects distinguishing biological regulators from their technological counterparts, Jones did not delve into the matter and, thus, did not address important distinctions between living systems, other natural systems, and artifacts.

### Dynamic stability and adaptivity

Other concepts related to regulation are dynamic stability and adaptivity. The former can be described as the capability to counterbalance the displacement of a system from a certain initial state provoked by a perturbation, ending up in the same final state (Rosen, 1970). Adaptivity, in turn, can be conceived as explained by Heylighen (2002), who argues that to adapt to a changing environment, a system needs a variety of stable states that are large enough to react to all disturbances but not so large as to make its evolution^3^ uncontrollably chaotic. As explained by him, around the middle of the twentieth century, researches from diverse backgrounds began studying phenomena characterized by inherent creativity, i.e., by the spontaneous appearance of novel structures or the autonomous adaptation of a system to a changing environment. These observations, along with concepts, methods, and principles they developed, gradualy coalesced into a new approach: a *science of self-organization and adaptivity*. Based on the behavior of dissipative structures as explained by the physical chemist Ilya Prigogine, the principle of self-organization enunciated by the cyberneticist W. Ross Ashby, and the principle of *order from noise*^4^ by Heinz von Foerster, researchers began to converge toward a science of adaptive dynamics. Central to this emerging framework was Ashby's principle, which proposed that a dynamical and nonlinear system (such as an ecosystem or the market), regardless of its type or composition, would always tend to evolve toward a state of equilibrium or an attractor (Heylighen, 2002). Using magnetization and Bénard cells as examples, Heylighen defines the concept of self-organization as the spontaneous creation of a globally coherent pattern out of local interactions of the system’s elements, without an external agent imposing it. This creation is made possible by the fact that the system is thermodynamically open, i.e., it shows a dissipative structure that absorbs matter and energy in a form of low entropy and exports them back to the environment with high entropy. By doing so, the system is able to reduce its own internal entropy while increasing the entropy of the environment, internally circumventing the second law of thermodynamics.

Self-organizing systems are considered to exhibit at least two traits or signatures that distinguish them from more traditional mechanical systems studied in physics and engineering (Heylighen, 2002). The first one, common to all self-organizing systems, is the absence of centralized control.^5^ As the parts contribute uniformly to distributed control in such systems, the responsibility for maintaining the organization is shared. Although different regions of a self-organizing system may be specialized for different tasks, no part of this system has overall control. In addition, these distributed organizations are redundant. This means that if one region of the system is damaged, it will be restored by the remaining regions, and the system’s overall functioning will be maintained, i.e., the system will show robustness or resilience, being relatively insensitive to perturbations (which in this case amount to errors).

Other contributors to the intrinsic robustness and resilience of self-organizing systems are their nonlinearity and the feedback relations among systemic components. Cause-and-effect relations in self-organizing systems are typically nonlinear, i.e., small causes can have large effects, while large causes can have small effects. This is due to circular or feedback relations among their components. Moreover, in self-organizing systems, the elements are structured and ordered to fulfill a certain function, namely, to maintain a particular systemic configuration in the face of perturbations (Heylighen, 2002). According to Heylighen, only self-organizing systems that perform closure can be self-maintaining and self-sufficient. This closure will be achieved when a chain or sequence of situations or events in a causal process becomes closed on itself (e.g., an event A causes B, which causes C, which causes D, which causes A). Therefore, the corresponding arrangement of the system will be continuously maintained or reproduced and will become largely independent from the environment. Despite the continuous exchange of matter and energy between the system and the environment, the organization will be determined internally, i.e., the system will be thermodynamically open and organizationally closed. However, notice that in this account, we are referring to closure of processes, not to closure of constraints, as put forward in the theory of biological autonomy and explained above. This difference is important because while closure of constraints is proposed to be exclusive to living systems, closure of processes is also found in physical and chemical systems (Moreno & Mossio, 2015).

The dynamic of self-organizing systems away from thermodynamic equilibrium is sensitive to changes in the environment. For instance, considering its dependence on an external source of energy, if that source disappears, the dissipative structure will disintegrate. Conversely, the surplus of energy allows the system to amplify ongoing processes and make it more dynamic and able to react (e.g., counteracting small perturbations by large reactions or sustaining positive feedback cycles for a much longer time). This makes the system much more powerful for developing, growing, or adapting to external changes. Instead of reacting to all perturbations by negative feedback that brings the system back to the same state of equilibrium, a far-from-equilibrium system is in principle capable of producing a much greater variety of regulating actions, leading to multiple stable configurations (Heylighen, 2002).

The second signature of self-organizing systems, attributed only to more complex adaptive systems such as organisms, is continuous adaptation to changing environments. A configuration of a system can be called *fit* if it is able to self-maintain or grow given the specific configuration of its environment. Otherwise, it would disintegrate spontaneously under the given boundary conditions. As Heylighen (2002) discusses, as different configurations can be compared in terms of their degree of fitness or likelihood of surviving under the conditions imposed by the environment, adaptation^6^ can be conceived as achieving a fit between the system and the environment. With large changes, the existing configuration becomes unstable, which may lead to its disintegration and the need to start the process of self-organization anew. This may not seem to be a great problem for self-organizing systems such as Bénard cells. However, for more complex systems such as organisms, ecosystems, and societies, adaptation becomes less trivial when the boundary conditions change since their eventual disintegration and the necessity to restart the process of self-organization would be much more costly.

Heylighen (2002) explores regulation in cybernetics as the control mechanism for minimizing deviations from a goal configuration in self-organizing systems. The process involves generating a variety of actions to handle potential perturbations and selecting the most suitable response. According to him, unlike mechanical control systems, self-organizing systems must autonomously develop these capabilities. Variety is fostered by the system’s operation in far from equilibrium conditions, while selectivity requires stable and limited configurations. Complex adaptive systems often reside on the *edge of chaos*, exhibiting self-organized criticality with power-law behavior. To select appropriate actions, a fitness criterion is crucial. Heylighen suggests using the environment as the criterion, but complex systems, such as organisms or minds, may mitigate risks by evolving internal models. These models act as a vicarious selector, internally testing potential actions virtually and, thus, enhancing reliability. However, Heylighen does not delve further into the subject of internal models, as it aligns more with centralized control than with self-organization.

As we noted above, there is a proposal that differentiates dynamic stability and feedbacks, as described by Heylighen (2002), from biological regulation (Bich et al., 2016). Biological regulation requires more complex mechanisms than those ensuring basic network stability. These are mechanisms that specifically rely on an asymmetry or hierarchical distinction between controller and controlled subsystems. Moreover, in this proposal, organizational closure is described as closure of constraints, not closure of processes. The constraints subject to closure, which are mutually dependent on each other, reduce the degrees of freedom of the processes and dynamics in which they act such that the system becomes capable of self-maintenance. In the next section, we will explain this proposed differentiation between control mechanisms in more detail.

### Biological regulation

Bich and colleagues (2016) argue that confusion exists in the use of the concept of regulation, which, according to them, conflates fundamentally distinct biological capacities. They propose, then, an approach in which biological regulation refers to a particular class of control capabilities that play a specific biological role and that are exercised by dedicated subsystems that do not perform constitutive metabolic functions. According to them, these regulatory subsystems give the organism the ability to deal with the effects of different perturbations by modulating its own internal dynamics, usually inducing a change to a new constitutive regime, selected among a diverse set of available ones. They intend, in these terms, to establish a conceptual distinction between two qualitatively different control mechanisms that contribute to biological robustness: (1) dynamic stability and feedbacks and (2) biological regulation. Since dynamic stability is realized collectively, it should be seen as a property distributed over an entire network of reactions and thus cannot be attributed to any single transformation or partial subset of transformations. This type of response depends only on the interaction among the components involved in reactions and control subsystems that already participate in the constitutive regime, without resorting to additional dedicated mechanisms. Among the mechanisms that contribute to maintaining the stability of the constitutive regime, we identify the aforementioned feedback loops. For these authors, negative feedback (or combinations of negative and positive feedbacks), despite enriching the dynamic behavior of a (bio)chemical system and increasing its potential dynamic robustness, should be considered as part of the constitutive regime. After all, they are dependent on direct coupling between subsystems that maintain stoichiometrically fixed interactions (which means that the component produced by a reaction is the starting component for the next reaction in a chain).

Regulation, by its turn, is explained by Bich and colleagues, as argued above, in terms of the activities of a subsystem other than the constitutive subsystem, albeit produced by the latter, whose function is to deal with internal and external perturbations. They consider that this subsystem performs a more elaborate and qualitatively different type of control, which mediates the effects of a perturbation by modulating (and possibly switching) the constitutive regime itself and/or its interaction with the environment, in order to produce a viable compensatory response compatible with changes in internal and external conditions. According to them, this capacity involves a different relationship architecture, as well as an increase in the overall organizational complexity of the system, compared to the distributed network that carries out the constitutive regime. Accordingly, regulatory control cannot be regarded, once explained in these terms, as a straightforward extension of the collective control enabling the dynamical stability of the constitutive regime. Rather, it demands a specialized, dedicated regulatory subsystem.

The concept of a regulatory subsystem is a key idea in Bich and colleagues’ approach and is crucial for the system to exhibit regulation according to their explanation. As Bechtel (2007, p. 90) explains,

[…] this subsystem is sufficiently independent of the dynamics of the controlled processes, and […] can be varied without disrupting these processes, but it is still able to be linked to parts of the controlled system [the regulated subsystem] so as to be able to modulate their operations.

In these terms, Bich and colleagues (2016, p. 253) argue that “a regulatory subsystem (R) needs to act freely from the constitutive regime (C) while at the same time being related to it: more precisely, it must (a) be produced by C and (b) be able to act on C”.

Another fundamental condition in this approach is that the activation and operation of the regulatory subsystem do not depend on the concentration, or on the variation in concentration, of its main components, i.e., this subsystem is stoichiometrically free from the constitutive system that gave rise to it (Griesemer & Szathmary, 2009). According to Bich and colleagues (2016, p. 254), “the action of R is triggered by specific changes in internal and/or external conditions, and the way it operates depends on its own internal configuration, not on the variation of its concentration levels”.

They argue that regulation enables a second-order control architecture that is dynamically decoupled from the constitutive system and is an important feature for increasing its robustness since it preserves the viability of the constitutive system through a mechanism that goes beyond its internal stability network. By robustness, they mean the ability of systems to maintain their functions and organization despite disturbances (Kitano, 2004). In addition, they highlight the relationship between regulation and homeostasis. According to these authors, homeostasis refers to the system’s ability to maintain some properties constant, while regulation usually brings about a change in the constitutive regime but maintains the identity of this system in a broader systemic context.

In short, Bich and colleagues (2016, pp. 256–257) present five main characteristics that allow for claiming that a system exhibits regulation as a response mechanism to a given perturbation:

1. Regulatory mechanisms/subsystems R are endogenously synthesized, i.e., they are produced by the constitutive regime C of the living system;
2. To be regulatory, R must be dynamically decoupled from C, which it regulates. This means that R, even if it is a product of C, operates at a different dynamical scale and under different stoichiometric requirements than C;
3. The activation of R is triggered by specific changes/perturbations P in either internal or external conditions, rather than by a change in the concentration of the components in R;
4. The functional role of R is to shift (either reversibly or irreversibly) between distinct constitutive/metabolic regimes C, C’, C’’…, C^n^ available to the system, depending on those variations in its internal or external conditions that trigger the activation of R;
5. The new metabolic/constitutive regimes C^i^ brought forth by R are capable of coping with the new conditions, extending the range of perturbations or stimuli to which the system may respond in a rapid and efficient way, as well as enriching the set of available functional dynamic behaviors.

For methodological reasons, Bich and colleagues (2016) focused on the regulation of metabolism in unicellular (prokaryotic) organisms to explain their distinct biological capacities. According to them, it should be easier to understand their fundamental characteristics and the role they play at the organizational level of a cell. However, regulation can surely be addressed, as they recognize, at many levels of description (from protocells to multicellular systems, or even in the ecological sphere). As stressed by these authors, at lower levels of description or organization, regulation is employed to describe similar but qualitatively distinct capacities than those instantiated in dynamic stability and feedbacks. The polysemy and often imprecision in the use of the term *regulation* and, thus, in the meaning ascribed to it may also extend to higher levels of description or organization, for instance, populations, communities, and ecosystems.

Bich et al. (2020) applied the organizational approach to regulation in multicellular systems by examining mammalian glycemia. They argue that while the standard feedback-loop model—which describes insulin and glucagon as controllers in a glucose-regulating circuit—effectively explains homeostatic behavior, it relies on a cybernetic machine analogy that oversimplifies biological complexity. This model localizes functions too neatly (e.g., “sensor,” “effector”) and ignores the distributed, hierarchical, and adaptive nature of organismal control. In contrast, the organizational framework characterizes regulation across multiple orders of functional constraints: first-order constraints (e.g., liver, muscle tissues) directly govern glucose metabolism; second-order constraints (e.g., hormones like insulin) modulate these metabolic processes; and higher-order constraints (e.g., neural signals) further adjust regulatory responses. This structure emphasizes integration and distributed control rather than linear feedback.

The idea of regulation at higher levels of biological organization played a central role in the debates on the cybernetic nature of ecosystems that divided ecologists across the 20th century. According to Hagen (1989), the presence of two markedly different intellectual perspectives during this period—holological and merological—largely contributed to the perceived intellectual immaturity of contemporary ecology. In the next section, we explore how the idea of regulation was addressed at higher levels of organization in this debate, aiming to elucidate the fundamental characteristics of regulation in ecosystems and its interplay with other concepts in the ecological discourse. Furthermore, to complement this analysis, we will consider how regulation is addressed in current studies in ecology, especially when considering the differences among types of control capacity proposed by Bich and colleagues.

## How regulation is addressed in the ecological literature

It is important to clarify that our purpose in addressing the contributions by Hagen is to highlight aspects related to regulation in ecological systems. These aspects are part of a broader theme in his work and are interwoven with other arguments. The texts by Hagen referenced here focus on the origins of ecosystem ecology and attempt to understand, through a historical account, why ecology is often considered an intellectually immature discipline or why it is distinct from other fields such as genetics and biochemistry. Our goal is to extract insights from these texts regarding how regulation in ecosystems has been approached in ecology, at which levels of organization it has been conceptualized—particularly at higher hierarchical levels—and in what ways. With this objective in mind, we aim to direct our efforts toward evaluating current studies in a more informed and deliberate manner.

### Regulation and its role in the debate on the cybernetic nature of ecosystems

G. Evelyn Hutchinson, a prominent ecologist in the 20th century, argued that there are two fundamental and complementary approaches in ecological research, namely, the merological and holological approaches (Hagen, 1989). The merological approach centers on populations of organisms, operating under the assumption that population phenomena determine the properties of higher levels of ecological organization. In contrast, the holological approach focuses on the flow of matter and energy through ecosystems without considering the organisms and populations that constitute such systems. Hagen considers that the approaches identified by Hutchinson reflected two broad intellectual standpoints analogous to visual perspectives, influencing how ecologists conceptualized relationships among natural units. According to Hagen, the holological perspective fundamentally provides a physiological view of nature, while the merological perspective offers a demographic view. These two distinct intellectual perspectives in ecology led to polarizations between extreme versions of each, contributing to a lack of conceptual unity and sparking debates, including discussions about the cybernetic nature of ecosystems.

Accordingly, ecologists, akin to the holological perspective, were inspired by physiology as a life science model to follow. Some of them focused on ecosystems and assumed a holological approach by studying the flow of matter and energy through food webs without considering the organisms or populations that make up the web (Hagen, 1992). The starting place for this perspective is identified by Hagen in Clements’ studies on ecological succession, which described communities as organism-like wholes and the process of succession as corresponding to their ontogeny, as a quasi-physiological process. Clements explained changes in vegetation stages in terms of successive processes of simple stimulus-response reactions between physical environments and organisms. The result of these successive interactions over time would express itself in the establishment of a climax community, a mature *organism* in equilibrium with its physical environment.

Despite criticisms of the treatment of biotic communities as superorganisms in Clements’ work, analogies with organisms continued to be used in ecology in subsequent years. For instance, the idea of community metabolism was largely developed out of earlier concepts of food chains and food webs by Charles Elton or of the more abstract trophic levels studied by the Odum brothers (Hagen, 1989). Both models strengthened the idea of interdependence between parts of the ecological system in the control of flows of matter and energy.

Although not supported by Elton, the *balance of nature* concept (Egerton, 1973) has sometimes been referred to as an older concept presenting the idea of self-regulation (Hagen, 1989). However, the concept most related to the idea of self-regulation in ecosystems is homeostasis. For Hagen, the idea that ecosystems are self-regulating entities was common among 1960s ecologists and has at least two roots in physiology. The first is found in cybernetics, a field that has been heavily influenced by physiology and proposes that animals and machines share common self-regulatory properties. One of the examples showing how cybernetics and physiology influenced the elaboration of the holological perspective in ecology lies in the advocacy by Hutchinson for negative feedback as a fundamental ecological concept. Howard T. Odum, one of Hutchinson’s students, followed this tradition, further developing an attempt to integrate these disciplines by seeking common mathematical expressions that could be applied to processes in machines, organisms, ecosystems, and human societies (Hagen, 1989). In the work of the other Odum brother, Eugene T. Odum, we also found an influence of physiology on ecological ideas related to self-regulation. Studying the variation in the heart rate of birds in different experimental situations in his doctoral dissertation, he also appealed to the concept of homeostasis, as formulated by Walter Cannon just seven years earlier. Cannon already intended to extend the concept of homeostasis beyond the field of vertebrate physiology, claiming that it could be used to explain processes in human societies. Similarly, Odum aimed at extending the domain in which that concept could be applied, focusing on ecosystems.

Although the holological perspective was most successfully applied in the study of ecosystems, it was also controversially used in the study of populations (Hagen, 1989). Until around the 1960s, it was common to see populations not as mere aggregations but as self-regulating entities with mechanisms to control their own growth. This view is exemplified by V. C. Wynne-Edwards, who explained population phenomena by drawing parallels with homeostatic systems. Wynne-Edwards suggested that populations can regulate their size through intricate negative feedback mechanisms triggered by behavioral signals or epideictic displays. This gave rise to criticisms based on his appeal to group selection, since under this reasoning, individuals would have to regulate their own reproduction to optimize overall population size. This was also one of the reasons for increasing polarization between the holological and merological perspectives.

In contrast to the holological perspective, the merological perspective starts from individual populations and uses a demographic approach to address ecological phenomena as the result of the proper dynamics of populations and species. Two different traditions applied a merological perspective, the individualistic and equilibrium approaches, which share important characteristics (Hagen, 1989). Both center around issues of distribution and abundance and generally define populations not as self-regulating entities but as mere aggregations defined in statistical terms. However, while the tradition of individualistic community ecology emphasizes the importance of stochastic processes and the independence of individual populations, the equilibrium community approach considers populations to be parts of tightly knit communities and assumes that demographic changes are mainly due to deterministic biotic interactions such as competition and predation. Communities are assumed to be in demographic equilibrium, i.e., gains by one population come at the expense of other populations. It is important to emphasize that from a merological perspective, populations are not understood as parts of a whole, and competition between two individuals is seen as nothing more than an interaction between independent individual organisms, while from a holological perspective, populations are parts of a biological system as a complex mechanical–organic entity. Competition would be, then, a kind of cybernetic or quasi-physiological function that stabilizes the entire community.

The polarization between the holological and merological perspectives in ecology gave rise to a series of controversies, including the debate on the cybernetic nature of ecosystems. On one side of this debate are those ecologists who argue that ecosystems are regulated by complex negative feedback mechanisms in which the flow of information transmitted through biogeochemical cycles and food webs controls the productivity and stability of the ecosystem, with disorder being something that cannot be ignored but may be viewed as abnormal or pathological. On the other hand, those who deny any similarities between organisms, machines, and ecosystems believe that substantial uncertainty is involved in ecosystem dynamics, thus demanding that the idea of stability be accepted with care and strongly supporting that disorder and randomness are normal rather than pathological aspects of ecosystem processes.

As Hagen (1989) argues, solving the conflict around the cybernetic nature of ecosystems was difficult, partially due to the imprecision of the stability concept. However, considering this controversy, we can also question whether stability is recognized as an important ecological property by different ecologists. Even though this is a difficult conflict to resolve, accepting analogies between homeostasis in organisms and self-regulation in ecosystems seems to depend, first, on accepting the philosophical point of view of the holological perspective.

After this brief overview of such a central debate in the history of ecology, we will consider in the next subsection how regulation is addressed in current ecological studies, considering the differentiation between types of control proposed by Bich and colleagues (2016).

### Regulation in current ecological studies

In this subsection, we present the results of a literature search for review studies on regulation in ecology. Using a simple search for reviews containing the terms regulat* and ecosystem* in their titles^7^, we identified a multiplicity of uses and meanings associated with the concept of regulation. The term was applied across both aquatic and terrestrial ecosystems and addressed various levels of biological organization, including molecular, organismal, population, community, ecosystems, and socioecological systems. We identified varied uses of the term “regulation”, including “nutrient regulation”, “mammal population regulation”, “top-down regulation”, “immunoregulation”, and references to “the theory of regulation”, among others. A total of 14 reviews and two textbooks were analyzed to assess the usage of the term (Supplementary material is available at: 10.5281/zenodo.17162280). Most of the reviewed cases fit into one of two categories: (i) regulation in the narrow sense (as defined by the organizational approach) occurring at the organismal level but resulting in or responding to ecological changes; or (ii) regulation in the broad sense, observed at the ecosystem level and involving mechanisms associated with dynamic stability and feedback loops. We briefly describe five cases sampled from our analytical *corpus* below, classifying them according to the different forms of control capacity proposed by Bich and colleagues (2016). The selected cases were drawn from the most cited reviews in our literature sample and include examples from standard textbooks. They span multiples levels of biological organization (from the subcellular to the socioecological level) and encompass diverse contexts, including bacterial, terrestrial, and aquatic ecosystems.

We begin by examining how regulatory mechanisms described at the subcellular level, such as quorum sensing in bacterial ecosystems (Fuqua et al., 1996), can propagate effects across higher organizational levels, including on ecological systems. These authors discuss *operons* (clusters of genes under the control of a single promoter, which works as a functioning unit) or *regulons* (clusters of genes controlled by the same regulatory gene that expresses a protein acting as a repressor or activator) involved in quorum sensing in marine bacteria. These gene clusters are found, for example, in the symbiotic marine bacterium *Vibrio fischeri*, in which they are also known as *lux genes* since they are involved in bacterial luminescence. During its growth, *V. fischeri* produces a diffusible compound designated *autoinducer*, which can accumulate at a concentration sufficient for the activation of lux genes. The *V. fischeri* membrane is permeable to the autoinducer, which is usually found at equal concentrations within cells and in their local environment. When *V. fischeri* are free-living in seawater, they are not luminescent, as they remain at lower densities, and the autoinducer is found at lower concentrations. However, when they occupy the environment of the light organs of fish or squids, *V. fischeri* reaches their highest densities, as does the concentration of the autoinducer that causes the bacteria to become luminescent through the activation of *lux* genes. Therefore, autoinduction can be viewed as a species-specific intercellular signaling system that allows *V. fischeri* to discriminate between the free-living, low cell density state and the host-associated, high cell density state, thus providing for the activation of luminescent genes during host association (Fuqua et al., 1996). This regulatory effect can lead to ecological consequences due to the interspecific relationship between fish or squid and *V. fischeri*. However, the regulatory effect is restricted to the organisms themselves and cannot be described in itself as ecological, as the new environmental conditions that trigger it only act as a perturbation to an individual (bacterium) level (and are not even part of one ecological regulatory mechanism itself). The mere aggregation of regulatory effects across multiple organisms is insuficient to make it a case of ecological regulation. The genes that allow for luminescence in *V. fischeri* are only activated when a certain concentration of the autoinducer is achieved due to the gathering of a number of individual bacteria. This is a clear case of regulation at the individual level, but the role of the ecological system (the population level) is only to establish the boundary conditions that allow for the perturbation (the autoinducer) to trigger the sensor of the regulatory subsystem in each individual bacterium. It should be noted that the components of the regulatory subsystem are parts of each individual bacterium. In the end, only the constitutive regime of each individual bacterium is affected as a consequence of their interaction. We could only attribute regulatory power at an ecological level if the relationships among the bacteria or between the bacteria population and the fish or squid were changed due to the luminescence.

Within the ecological literature surveyed, regulation is further conceptualized as a process underpinning homeostasis, that is, an organism’s ability to maintain internal stability (e.g., salt/water balance or body temperature) despite fluctuations in its external environment. For instance, Robert E. Ricklefs and Rick Relyea, in his textbook “*Ecology: The Economy of Nature*” (2014), offered some examples of these regulatory mechanisms. One of them is the second case we would like to mention, which concerns what happens to some fish species during their reproductive migrations. These migrations can take two paths: fish that live most of the time in saltwater environments and migrate to freshwater environments during the reproductive season (anadromous species) and fish that perform opposite movements (catadromous species). In both situations, there are physiological changes that allow them to deal with the excess or restriction of solutes in the external medium. Salmon (*Salmo salar*), for example, are born in freshwater environments, and as they grow, they acquire characteristics that allow for acclimation to saltwater environments by means of osmoregulatory mechanisms, which change from a hyperosmotic regulator (where there is active reabsorption of NaCl in proximal and distal renal tubules) to a hypoosmotic regulator (where there is active secretion of MgSO_4_ in those tubules). These regulatory mechanisms involve hormonal modulation that changes gene expression, which, in turn, alters the composition of the ion channels responsible for the absorption or removal of ions that could hinder the survival of the organism. As in the case of *Vibrio fischeri* described above, this also seems to be a case of purely organismal regulation in which environmental factors act solely as perturbations and, in turn, lead to consequences at the ecological level, while not being an ecological regulatory mechanism per se.

At the community level, regulation can be related to the control of population growth through negative feedback mechanisms triggered by density-dependent factors (Begon et al., 2005; Ricklefs & Relyea, 2014). As their designation suggests, these factors control population growth through the intensification of their effects with increasing population density (e.g., the effects of scarce food supply or space to live or the effects of predators, parasites, and diseases that become stronger in denser populations). The third case we derived from our review study provides an example concerning the relationships between population regulation of mammal species, keystone processes, and ecosystem dynamics (Sinclair, 2003). Sinclair describes how bottom-up processes such as food shortages (or some other resource) or top-down processes such as predation regulate animal populations through negative feedback mechanisms (involving density-dependent factors). He also explains the effects that differences in mammalian body size have on the strength of the regulation process. According to him, there is an indication that, in small mammals, density dependence is stronger at low numbers. In turn, density dependence in large mammals is stronger at high population numbers, which may produce a more rapid decline in their per capita rate of increase. This means that at low population numbers, large mammals have weak density dependence to compensate for disturbances caused by climate, predators or competitors, and thus, they are vulnerable to extinction under these conditions. The term regulation is used in this case to refer to a process that keeps a population near demographic equilibrium, as has been proposed from the merological perspective of equilibrium communities. In this case, the regulatory mechanism is not carried out by any specialized subsystem but rather relies on the tight coupling between various distributed parts of the constitutive regime of the ecological system (e.g., resources, prey, predators, and so forth); this can be regarded as an example related to dynamic stability and feedbacks.

A fourth example we found in the review papers surveyed concerns the role of the condensed tannins of some plants in regulating soil nutrient availability through different effects on ecosystems. For instance, Schweitzer et al. (2008) used condensed tannins (CTs) as an example to discuss how the production of this polymer by species from the genus *Populus* has the capacity to structure communities. They examined the various mechanisms for CT expression and their ecological consequences in streams and forests. These authors highlighted four aspects related to CT that explain its effects on nutrient regulation in the ecosystem: (i) the plant-specific concentration of CT varies among individual species and genotypes; (ii) a large within-plant variation in CT occurs due to ontogenetic stages, tissue types, and phenotypic plasticity in response to the environment; (iii) CT has an inconsistent effect on plant-herbivore interactions, except for organisms that use woody tissues; and (iv) CT in plants consistently reduces rates of litter decomposition (aquatic and terrestrial), alters the composition of heterotrophic soil communities (and some aquatic communities), and reduces nutrient availability in terrestrial ecosystems. Again, we are dealing in this case with the propagation of effects in an ecological network of constitutive constraints, or, in Bich and colleagues’ terms, with just dynamic stability and feedbacks.

Finally, a fifth example is found in the description of the role of ecosystems in regulating climate, as well as soil, water, and air quality. For example, Smith et al. (2013) examined the role of ecosystems in delivering these regulatory ecosystem services in a socioecological context (in the UK) to identify the co-benefits and trade-offs resulting from managing ecosystem processes that support these services. These authors summarize the ecosystem processes responsible for each of these regulatory ecosystem services and explain how they are regulated by ecosystems. For example, ecosystem processes such as carbon storage, heat and moisture transfer, nitrous oxide (N_2_O) and methane (CH_4_) emissions, aerosol formation, changes in albedo, and microclimate regulation together contribute to providing climate regulation ecosystem services. All these processes are carried out by the activity of biodiversity, such as plant species that store carbon during their growth and transfer heat and moisture to the atmosphere via evapotranspiration, which ultimately contributes to climate regulation. However, as Smith and colleagues are analyzing a socioecological context, they also include the human activity of managing ecosystems among the elements responsible for contributing to the production of the regulation service. Carbon storage for hundreds of years derived from the extraction of wood used in housing is one of the examples given by the authors in this respect. In this case, although we should consider other types of norms inherent to socioecological systems and sociopolitical contexts (such as human social norms), the term *regulation* is primarily used in reference to relations between ecosystems and regulatory processes. Therefore, this is yet another example of processes conducted through the tight coupling among various distributed parts of the constitutive regime of a socioecological system (including humans, vegetation, and atmosphere) that can be interpreted as an instance of dynamic stability and feedbacks.

As in organismal and cell biology, the different uses of the concept of regulation in ecology can generate important inaccuracies in its definition and understanding, in addition to the ambiguities found in other related concepts. We were not able to find any study that proposed a specialized subsystem devoted to regulation in an ecological system, which could be described as being dynamically decoupled from the network of relations constituting the latter or, at least, as not working through the propagation of perturbations through this network. This means that we could not find an approach to regulation in the ecological literature, as proposed by Bich et al. (2016), based on the theory of biological autonomy.

## Discussion

The concept of regulation is typically defined as the capacity of biological systems to maintain internal conditions within a relatively constant or optimal range despite external fluctuations, as exemplified by frameworks emphasizing homeostasis and dynamic stability. The cases identified in our literature search reflect this definition by primarily detailing regulatory mechanisms at the organismal level that respond to or induce ecological changes, alongside mechanisms of dynamic stability and feedback loops operating at higher levels of biological organization (e.g., populations, communities, and ecosystems). Although it was not captured in our literature search, there is a well-developed body of work on ecological regulation in the context of population dynamics that critically associates regulation with the dogma of the “Balance of Nature”, as the merological perspective of equilibrium communities discussed by Hagen (1989). A pivotal analysis of this tradition is found in Cooper’s (2003) “*The Science of the Struggle for Existence*,” which examines two contrasting arguments inherent to this dogma. The first posits that populations are inherently regulated by density-dependent biotic factors (e.g., competition), maintaining stability and equilibrium through evolutionary processes. The second perspective, exemplified by White’s work on psyllid insects, argues that persistence often results from stochastic, abiotic factors (e.g., nutrient distribution or environmental variability), emphasizing contingency and nonequilibrium dynamics. Criticizing both views, Cooper asserts that regulation is often misapplied through circular or a priori reasoning, such as equating phenomenological patterns (e.g., population return tendencies) with causal mechanisms like density dependence. He concludes that questions of regulation are fundamentally empirical and cannot be resolved by conceptual arguments alone, urging a move beyond dogmatic assumptions toward evidence-based, context-dependent explanations.

The organizational theory of ecological functions has faced criticism for its apparent commitment to equilibrium ecology, particularly regarding its reliance on organizational closure as a criterion for functional assignment. Critics such as Lean (2021) argue that this approach assumes stable, self-sustaining systems with cohesive interactions among species, a view taken to be aligned with equilibrium ecology but inconsistent with non-equilibrium perspectives, which emphasize historical contingency, dispersal, and stochastic processes in the formation of open, dynamic communities. However, proponents of this theory argue that organizational closure does not require equilibrium assumptions. They emphasize that, even in nonequilibrium systems, functional organization can emerge from interactions between local species once communities have formed, regardless of whether their formation was driven by deterministic or stochastic processes, or both (see Vellend, 2016). The organizational approach provides a clear criterion for identifying organizational closure in ecological systems: in a closed organization of constraints, not all constraints relevant to the system’s self-maintenance are included within organizational closure but only constraints that are both enabling and dependent. Several constraints that are enabling but not dependent, or contrariwise, connect an ecosystem to other systems, and make it possible accommodate different degrees of openness and contingency. Accordingly, the requirement of organizational closure in ecosystems brings much less restriction to functional ascription on an organizational basis than it might seem at first sight (El-Hani et al., 2024; Saborido, El-Hani & Moreno, in press).

Based on this flexibility, proponents of the organizational theory have recently advanced a series of hypotheses related to cluster configurations within ecological interaction networks (Pinto Leite et al., in review ). These hypotheses range from highly integrated “organized” clusters, where constraints exhibit mutual dependence and closure (allowing for the ascription of organizational functional and intrinsic teleology related to self-maintenance through closure), to “structured” clusters with local ecological interactions contributing to community persistence (requiring alternative theories to ground functional ascriptions, such as persistence enhancing propensity account, see Dussault & Bouchard, 2017), and finally, mere “aggregates” of populations forming just assemblies with no higher-level community persistence (where ecological functions can only be ascribed below the community level, to populations or organisms). This nuanced framework recognizes that ecological systems vary in how they are structured and that functional explanations must be adjusted accordingly, whether through organizational, dispositional, or etiological theories (in the case of ecological functions in the form of organismal activities, see Dussault, 2022).

While studies by proponents of the organizational approach argue for greater theoretical emphasis on control mechanisms due to their critical role in biological system self-maintenance, their focus has been circumscribed to cellular and organismal levels (Bich et al., 2016, 2020; Bich & Bechtel, 2022). It is important not to overlook that although organisms and ecosystems are both biological systems, differences in their fundamental properties can lead to distinct ways of achieving control in response to perturbations.

The five criteria delineated by Bich et al. (2016) can help to identify putative cases in which a regulatory subsystem might be found in a system. Criterion two is of special relevance when applying this approach to ecological systems. At first glance, the example of chemotaxis in bacteria given in the original paper by Bich and colleagues might give the impression that differences in stoichiometric requirements are mandatory. The concentrations of the components of R (signaling molecules that reverse the flagellar rotation) do not change; they are continuously produced by the cell, and their quantity does not increase when the regulatory subsystem is activated. This is the same as saying that there is a certain quantity of matter that is allocated exclusively to R at all times. This is obviously a trade-off that the cell can afford: matter and energy that are useful for maintaining its own existence are allocated not as part of constitutive constraints but as regulatory constraints that are contingently activated but allow the system to deal with many situations that threaten the cell’s existence. Moreno and Mossio (2015) call this a second-order closure: the subset of constitutive constraints generates the subset of regulatory constraints, while the latter allows the existence of the former in a wider array of situations: in the end, each subset is enabling of as well as dependent on the other, and thus, they realize closure when considered together. This constitutes an obvious selective advantage: the trade-off is justified, as each individual organism that instantiates this organization has more chance to survive than its competitors with no or less efficient regulatory constraints. Thus, as an organization evolves, regulatory subsystems become an important part of it, as they are necessary for the organisms in each generation to fulfill the end of keeping their own existence.

We submit that ecological systems cannot afford the same trade-off as a cell or organism that allocates matter exclusively to a regulatory subsystem R. There is no such thing as resources that are used only contingently in ecological systems, and these systems are typically conceived as not evolving through natural selection in a way that could justify the existence of such a trade-off (or, at least, there is no widely accepted account on how they could evolve by natural selection). Thus, as far as a comparison between cells or organisms and ecological systems can go, one would be tempted to conclude that it is impossible to exist regulation, as defined by Bich and colleagues, in ecological systems.

Yet, this conclusion may be too hasty. Instead, we can ask whether it is truly necessary to elaborate the author’s second criterion in the sense that a certain quantity of matter should be allocated exclusively to R at all times. This criterion can be reformulated, opening the possibility that ecological systems exhibit more than just dynamic stability and feedback mechanisms, and may be worthy subjects for applying Bich and colleagues’ framework.

Crucially, investigating this possibility requires a clear method for differentiating constitutive constraints from regulatory ones, which hinges on deciding about the correct level of analysis: should we consider organisms themselves as constraints? Or their parts? Or even entities above the level of organisms, such as populations? The bromeliad model presented by Nunes-Neto and colleagues (2014) ascribes the role of constraints to organisms as wholes, due to the activities related to feeding and nutrition they perform. Focusing on feeding activities is clearly appropriate when describing constitutive constraints: they are the way organisms harness the flow of matter and energy in such a way that enables the persistence of the very same organisms that act as constraints in a first-order closure. Finding regulation, in turn, demands identifying activities that organisms perform (constantly or occasionally) that do not contribute to first-order closure. The complication resides in the question: “What kinds of activities indicate a given organism is acting as a first-order or a second-order constraint?”. Or even “How to separate these activities in time, when modelling regulation?”.

Due to space limitations, we will not elaborate this topic in detail here, but some initial thoughts are in order. Finding a suitable case for ecological regulation, we submit, involves identifying activities that are not related to feeding but can lead to changes in the operation of first-order closure. In order to separate them in time (and, when relevant, in space), these activities should be primarily thought of as being performed by parts of organisms, not organisms as wholes. Additionally, this helps ensure (in a first approach) that we do not wrongly characterize regulatory phenomena that occur solely at organismal level as ecological regulation: an ecological regulatory subsystem should be composed of parts of different organisms. As a candidate, we indicate the case of herbivore-induced plant volatiles (HIPVs). These volatiles are produced and released into the air by some plants when they are attacked by herbivore insects and attract predators and parasitoids that can suppress the herbivore population (Aljbory & Chen, 2018). Following Bich and Bechtel’s (2022) approach on how to think about regulatory subsystems, the plant’s parts that detect herbivory acts as sensory constraints, detecting the perturbation; the plant’s parts that produce and release the HIPVs, and the predator and parasitoids’ parts that detect the HIPVs acts as intermediate constraints, connecting the sensory to the effector constraints; the predator and parasitoids’ parts that change their behavior such that they look for the HIPVs sources are their effector constraints, acting on the relevant constitutive constraint. The predator and parasitoids’ parts involved in their hunting and feeding activities would, then, be the constitutive constraints modulated by the regulatory subsystem. A detailed analysis of this case, however, is a matter for another work.

Last but not least, it is also important to consider that, in a different vein, new mechanists have been exploring control mechanisms, i.e., mechanisms that are external to and act upon other mechanisms responsible for the basic metabolic and motor activities of organisms (Winning & Bechtel, 2018). While the theory of biological autonomy and, generally speaking, the autonomy tradition as a whole focus on understanding biological systems as self-maintaining closed networks of constraints, the new mechanism tradition focuses on understanding how organized parts interact in order to perform activities required to generate phenomena that biological systems exhibit (Bich & Bechtel, 2021). However different, Bich and Bechtel (2022) argue that these traditions are not incompatible and complement each other, with regulatory constraints and control mechanisms providing a theoretical bridge between them. As ecology bears much influence from mechanist ideas, this bridge should not be ignored in future conceptions of regulation models in ecological systems.

Although Bich and colleagues (2016) limit their criticisms about inaccuracies in the meaning of regulation to the level of organisms, we extend those same criticisms to the study of ecological systems. In this paper, we endeavor to extend their analysis as well. In our view, this not only has the potential to bring philosophical and theoretical advancements to ecology, as noted by Bich et al. (2020) but also to make significant contributions to discussions about other concepts used in ecology and the environmental sciences, such as ecosystem health (Sfara & El-Hani, 2023). Additionally, it can also help to solve ambiguities concerning terms used in the ecological literature, such as homeostasis and dynamic stability, thereby highlighting their different meanings and the requirements for their precise use. Finally, the proposed distinction between different types of control may inspire new explanations that highlight previously overlooked aspects of ecosystems and should be encouraged in ecological research.

## Footnotes

1 This theory has been built based on a biological and philosophical tradition that comes back to authors such as Immanuel Kant, and his idea that biological systems are self-organized and has “natural purposes”, as means and ends of themselves; Georges Cuvier and his principle of the “conditions of existence”, which claims that there is a strong coordination among the different parts of each living being that makes it possible its existence as a whole; Claude Bernard and his approach to biological systems as organized entities whose parts are interdependent and mutually generative; Ludwig von Bertalanffy, who conceived living beings as thermodynamically open systems continuously exchanging energy and matter with their surroundings; Jean Piaget, who highlighted the connection between closure and organization, and tried to integrate them into a single coherent picture as two inherent dimensions of biological systems, namely, their thermodynamic openness and organizational closure; Maturana and Varela and their account of biological organization based on the concept of autopoiesis; Robert Rosen and his proposition that the distinction between closure and openness should be grounded on a distinction between efficient causes and material causes; and, finally, Stuart Kauffman, who argued that biological organization implies a circular relationship between work and constraints, in the form of a “work-constraint (W-C) cycle” (Moreno & Mossio, 2015; Mossio et al., 2016; see the original works for more details).

2 We model two cases of regulation in ecological systems (*sensu* Bich et al., 2016) in Carvalho et al. (in preparation).

3 In order to avoid any ambiguity: here, Heylighen is not referring to evolution as related to changes that lineages suffer over time, but to changes of states in a particular system over the time of its individual existence.

4 This principle describes, as discussed by Heylighen (2002, p. 3), a paradox: “the larger the random perturbations (“noise”) that affect a system, the more quickly it will self-organize (produce “order”)”. He further explains this idea as follows: “[…] the more widely a system is made to move through its state space, the more quickly it will end up in an attractor. If it would just stay in place, no attractor would be reached, and no self-organization could take place” (p. 3).

5 Examples of self-organizing systems without centralized controls are found in the most diverse domains, e.g., neural networks, laser production, and the spontaneous creation of collective behaviors in animals, as we see in flocks of birds, schools of fish, swarms of bee, or herds of sheep.

6 It seems to us that Heylighen (2002) uses at least two meanings for the concept of adaptation in his explanations. When he says, for example, that “[…] adaptation can be conceived as achieving a fit between system and environment (p. 15)”, or that “Systems may be called adaptive if they can adjust to such changes while keeping their organization as much as possible intact (p. 15)”, he seems to refer to a notion of adaptation in a physiological sense, and, thus, his statements seem to relate to Bernard’s notion of conservation of the internal *milieu*. However, when he exemplifies adaptations in ecosystems later in his text, claiming that, “When the circumstances in an ecosystem change (e.g., the climate becomes drier), those species or varieties in the ecosystem that are better adapted will become more numerous relative to those that are less, until the balance is restored (p. 16)”, he advances an argument that can also be interpreted in terms of the notion of adaptation in an evolutionary sense, i.e., a notion anchored in natural selection as explained in Darwin’s theory of evolution. We do not intend to make an in-depth analysis of this ambiguity we identify in Heylighen, but we consider it important to point here.

7 For more details about the literature search methods and the construction of the spreadsheet with our search results, please refer to ‘Text S1. Methods used in the literature search and the construction of the spreadsheet.’ in the supplementary material.
