# Correction: Regulation in ecological systems: an overview

**DOI:** 10.1007/s40656-025-00715-0

**Published:** 2026-01-09

**Authors:** Clarissa Machado Pinto Leite, Ítalo Nascimento de Carvalho, Jeferson Gabriel da Encarnação Coutinho, Charbel N. El-Hani

**Affiliations:** 1 Institute of Biology, Federal University of Bahia, Salvador, Brazil; 2 National Institute of Science and Technology in Interdisciplinary and Transdisciplinary Studies in Ecology and Evolution, Salvador, Brazil; 3 Bahia Federal Institute of Education, Science and Technology, Salvador, Brazil

**Correction to: History and Philosophy of the Life Sciences (2025) 47:58** 10.1007/s40656-025-00707-0

In this article the author’s name ‘Jeferson Gabriel da Encarnação Coutinho’ was incorrectly written as ‘Jeferson Gabriel Encarnação da Coutinho’.

The original article has been corrected.

